# Improving the assessment of communication competencies in a national licensing OSCE: lessons learned from an experts’ symposium

**DOI:** 10.1186/s12909-020-02079-4

**Published:** 2020-05-26

**Authors:** Matteo Monti, Christina Klöckner-Cronauer, Stephanie C. Hautz, Kai P. Schnabel, Jan Breckwoldt, Noëlle Junod-Perron, Sabine Feller, Raphael Bonvin, Sören Huwendiek

**Affiliations:** 1 Medical Education Unit, Faculty of Biology and Medicine, University of Lausanne, Rue du Bugnon 21, 1011 Lausanne, Switzerland; 2 Institute of Medical Education, Department of Assessment and Evaluation, University of Bern, Bern, Switzerland; 3 Student’s Deanery, Medical Faculty, University of Zürich, Zürich, Switzerland; 4 Division of Primary Care, Geneva University Hospitals, Geneva, Switzerland; 5 Unit of Development and Research in Medical Education, Faculty of Medicine, Geneva, Switzerland; 6 Medical Education Unit, Faculty of Sciences and Medicine, University of Fribourg, Fribourg, Switzerland

## Abstract

**Background:**

As the communication competencies of physicians are crucial for providing optimal patient care, their assessment in the context of the high-stakes Objective Structured Clinical Examination (OSCE) is of paramount importance. Despite abundant literature on the topic, evidence-based recommendations for the assessment of communication competencies in high stakes OSCEs are scarce. As part of a national project to improve communication-competencies assessments in the Swiss licensing exam, we held a symposium with national and international experts to derive corresponding guidelines.

**Methods:**

Experts were invited on account of their recognized expertise either in teaching or assessing communication competencies, or in conducting national high-stakes OSCEs. They were asked to propose concrete solutions related to four potential areas for improvement: the station design, the rating tool, the raters’ training, and the role of standardized patients. Data gene.rated in the symposium was available for analysis and consisted of video recordings of plenary sessions, of the written summaries of group work, and the cards with participants’ personal take-home messages. Data were analyzed using a thematic analysis approach.

**Results:**

Nine major suggestions for improving communication-competencies assessments emerged from the analysis and were classified into four categories, namely, the roles of the OSCE scenarios, rating tool, raters’ training, and simulated patients.

**Conclusion:**

In the absence of established evidence-based guidelines, an experts’ symposium facilitated the identification of nine practical suggestions for improving the assessment of communication competencies in the context of high-stakes OSCEs. Further research is needed to test effectiveness of the suggestions and how they contribute to improvements in the quality of high-stakes communication-competencies assessment.

## Background

Good physician-patient communication is crucial for optimal patient care, as evidenced by its positive impact on outcomes, such as patients’ health, compliance, trust, and related healthcare costs [1–3]. On the other hand poor communication accounts for a significant portion of patients’ complaints [4]. As is the case in many countries, the Swiss framework for undergraduate medical training requires medical faculties to provide communication-skills training and to conduct assessments of communication competencies throughout the undergraduate curriculum [5, 6]. Assessment of physician-patient communication competencies can be performed through direct observation of interaction with real patients, rating of encounters with standardized patients, rating of interactions recorded on audio- or videotape, patient or multisource questionnaires [7, 8]. Direct observation of clinical encounter with standardized patients is widely used by medical schools and residency programs, since it provide evaluation of communication and interpersonal skills in a high fidelity and authentic setting [7] Since 2011, the clinical skills component of the Swiss Federal Licensing Exam (FLE), which consists of a 12-station Objective Structured Clinical Examination (OSCE) [9], has been used for the systematic assessment of communication competencies at all stations. Alongside the USA and Canada, Switzerland is one of the few countries in the world that introduced the OSCE format as part of the national licensing exams. Despite the recognized appropriateness of the OSCE format for assessing complex communication skills [10, 11], the reliable and valid assessment of these competencies poses challenges. For instance, the multiplicity of frameworks developed over the last decade to describe what constitutes “good physician-patient communication” and the “communication tasks” to be accomplished during medical encounters [12–14], has led to the development of many assessment instruments for communication competencies without an agreed-upon gold standard for the OSCE setting [15–17]. Furthermore, compared to other clinical skills, communication competencies seem to be harder to assess reliably [18]. Indeed, research shows low *inter-case* reliability as a consequence of high context-specificity, and low *inter-rater* reliability, which is probably intrinsic to the subjective nature of an assessment of communication [18]. During the clinical skills component of the FLE, communication competencies are assessed at every station using a global rating scale derived from the Analytic Global OSCE Ratings developed by Hodges and McIlroy [19, 20], which measures four dimensions: (a) addressing the patient’s needs, (b) structure of the conversation, (c) verbal expression, and (d) nonverbal expression. Several aspects were considered in the selection of this instrument: (1) differences among Swiss medical schools in terms of their instructional models and assessment tools for communication competencies; (2), ease of use by examiners without a need for extensive training; (3) sufficiently general to ensure students of different medical faculties are not placed at a disadvantage, and, (4) the assessment of communication competencies should be completed in less than 2 min. In the Swiss FLE context, this scale showed over the years good internal consistency among the four dimensions, with a Cronbach’s alpha ranging from 0.85 to 0.90. However, internal quality analysis (data not published) suggested that the scale’s high internal consistency might have been due to the raters’ inability to differentiate between the four dimensions of this scale.

These reflections led to the question of how to improve assessments of communication competencies in the Swiss FLE. The Swiss Federal Office of Public Health funded a national project in 2014 to address the challenge of improving the assessment of communication competencies. The first step of the project consisted of a nationwide survey; collecting data from instructors and students on how Swiss medical schools train students to apply communication skills [5]. The second consisted in a literature review and a second survey exploring perspectives suggested by the FLE candidates, raters, and communication-competency instructors on how to improve assessments of physician-patient communication for the Swiss FLE. This allowed us to identify four potential areas for improvement: (1) the station design, (2) the rating tool, (3) the raters’ training, and (4) the role of standardized patients (SPs). The next step was aimed at developing concrete measures pertaining to these areas by organizing a symposium with international experts. This article reports and discusses the main themes that emerged from this symposium. Given the scarcity of evidence-based recommendations for physician-patient communication assessment in the high-stakes licensing OSCE [12, 18, 21–23], we believe this article may offer practical suggestions to all people involved in the organization of this type of OSCE.

## Methods

### Participants

Twenty-nine communication and assessment experts met for a 2-day symposium held in February 2016 in Bern, Switzerland. The four international experts (from Canada, Italy, the Netherlands, and the United Kingdom) were recognized for their expertise and research activity either in teaching or assessing physician-patient communication competencies, or in conducting national high-stakes OSCEs. The twenty-five experts from the five Swiss medical schools were communication instructors and researchers, members of the experts’ group in charge of the conceptualization and quality improvement of the national OSCE, and faculty members in charge of the communication-competencies curricula.

### Symposium delivery and methods

The symposium started with a presentation of the above-mentioned national survey. Each of the international experts then presented their perspective on the assessment of communication-competencies. Participants were then divided into small groups and discussed possible improvements regarding the four predefined areas (station design, rating tool, raters’ training, and SPs’ role). At the end of every day, each group presented and discussed its achievements in plenary sessions. The last part of the symposium consisted of a plenary discussion of the main lessons learned. Participants were asked to propose and discuss concrete implications for the future communication skills assessment within the Swiss Clinical Skills-FLE. The plenary sessions were video-recorded. At the end of the symposium, we asked each participant to write down a personal take-home message.

### Data analysis

Data generated in the symposium were available for qualitative analysis and consisted of video recordings of the two plenary sessions (2 h 55 min), of the written summaries of group work, and of the cards with personal take-home messages. Data were analyzed using a five-phase thematic analysis approach [24]. Thematic analysis is a flexible approach to the analysis of different types of qualitative data [24].. Since analysis began several weeks after the symposium and data were derived from different sources, the use of thematic analysis seemed to us an appropriate method for analyzing and organizing the available material, thus minimizing the risk of memory distortions. All authors of this manuscript participated in the symposium. CK, MM, RB, SH, and KS conducted phases 1 to 3 (i.e., they became familiar with the data, generated codes, and identified themes). All authors were involved in reviewing and labelling the themes (phases 4 and 5). Discrepancies were discussed until consensus was achieved. Themes were categorized into the four predefined areas. A theme was reported if it was addressed in all three settings (plenary sessions, work groups, and take-home messages), as indicators of its importance to the participants.

## Results

The thematic analysis helped us highlight nine major themes, which were classified according to the four pre-established areas (Table 1). Statements written in quotation marks and italics correspond to the verbatim transcription of excerpts from the video recordings.

**Table 1:** Major suggestions identified through the thematic analysis

***How can the design of OSCE stations improve the assessment of physician-patient communication competencies?***	
1. Develop scenarios aimed at measuring the adequacy of examinees’ responses to pre-specified emotional cues	
2. Develop scenarios with a main focus on specific communication situations	
3. Involve communication experts in the in the development of the OSCE stations	
4. Monitor the balance between authenticity and standardization	
***How can the rating scale improve the assessment of physician-patient communication competencies?***	
5. Ensure the presence of items that capture case-specific communication outcomes	
6. Use a global rating scale for the assessment of general communication competencies	
***How can simulated patients contribute to the assessment of physician-patient communication competencies?***	
7. Include an additional assessment of communication by SPs	
8. Adapt SPs’ training to the new stations for the assessment of communication competencies	
***How can the raters’ training be improved?***	
9. Adapt the raters’ training to the new stations dedicated to the assessment of communication competencies	

### How can the design of OSCE stations improve the assessment of physician-patient communication competencies?

The variety of contexts to which candidates are exposed during the FLE includes a large sample of situations. However, to better discriminate the levels of communication competence, participants also suggested designing stations with a specific focus on communication:

1. **Scenarios measuring the adequacy of examinees’ responses to a pre-specified patient situation with emotional distress**

Participants suggested “*enriching some of the traditional stations with specific communication challenges, for example, by introducing specific emotional cues or concerns in the OSCE case”*. These cases aim to measure the appropriateness of the examinee’s responses to emotional distress portrayed by the SP. “*The emotional cues or concerns could be expressed verbally or non-verbally and should be related to the medical problem”* or its perceived consequences. A suggestion was made to use the Verona Coding Definitions of Emotional Sequence (VRCoDES) [25, 26] as a framework for the development of such scenarios, and to develop a measure to assess the appropriateness of the examinee’s response. Participants anticipated a potential pitfall of this approach “*if the examinees expected an emotional agenda in every patient encounter”,* and consequently, would adopt a non-authentic, test-induced communication style. Hence, participants suggested limiting the number of stations with specific emotional stimuli and varying the types and intensity of the emotional states to be portrayed (e.g., anger, sadness, or anxiety).

2) **Scenarios with the main focus on specific communication situations**

Participants suggested “*developing specific OSCE scenarios where communication would be the core of the clinical encounter”*. For such stations, specific communication situations (e.g., breaking bad news or a motivational interview) were proposed. To ensure content validity, participants proposed the development of OSCE scenarios that were built on validated communication models (e.g., the SPIKES-model for breaking bad news) [20]. The importance of selecting communication models corresponding to those taught during medical training was stressed. Participants also suggested creating *“a platform for the communication instructors from the five medical schools to exchange information and impressions about such models”*.

3) **Involvement of communication experts in the development of the OSCE stations**

Given the specificity of the communication models used in the new OSCE stations, the participants anticipated that “*not all clinicians would be familiar with these concepts”*. Therefore, a recommendation was made to pair communication experts with clinical experts for the case-writing process.

4) **Balance between authenticity and standardization**

The participants stressed the “*need to strive for high levels of authenticity and standardization in the context of high-stakes assessments*”. They pointed out the difficult trade-off between these two characteristics. For example, if, in the attempt to achieve higher standardization, case writers develop very detailed SP scenarios, the SPs would have less flexibility in adjusting their role-plays to correspond to the quality of the examinee’s communication. “*A one-fits-all response of the SP to all examinees’ interactions would, therefore, decrease the authenticity of the experience*”. For this reason, some participants proposed allowing some flexibility in the SP’s portrayal, based on whether or not the examinee adopted the expected communication attitude.

### How can the rating scale improve the assessment of physician-patient communication competencies?

Medical content (medical history, physical examination, and management) is actually assessed using a case-specific checklist, while communication competencies are assessed at all stations using a global rating scale derived from the Analytic Global OSCE Ratings [19, 20].

5) **Ensure the presence of items that capture case-specific communication outcomes**

Participants stressed the importance of “*ensuring that items on the checklists and the global rating scale systematically capture the specific communication goals of each station”*. From the perspective of a licensing examination, participants pointed out how it might be more meaningful to assess the “outcome of the encounter” (e.g., “*Did the candidate obtain relevant patient information?” “Did the candidate ease the patient’s fear?” or “Did the patient understand the candidate’s explanation?*”), than the technique the candidate used to achieve the results (e.g., “*Did the candidate use the correct communication technique or model?*”).

6) **Having a global rating scale for the assessment of general physician-patient communication competencies**

Given the concerns about the inability of raters to differentiate between the four dimensions of the Analytic Global OSCE Ratings, participants proposed a focus on familiarizing raters with the scale, rather than changing it. To achieve this, they proposed “*having all faculty members in the undergraduate curriculum use this scale for both summative and formative assessments”*.

### How can simulated patients contribute to the assessment of physician-patient communication competencies?

7) **Additional assessment of communication by SPs**

Some participants suggested involving SPs in the evaluation of communication competencies in order to achieve a more accurate discrimination of good communication. They argued that “*assessments by SPs could be complementary to those of physician raters because they perceive other dimensions of communication”*. The feasibility of this proposal depends on the time interval between the stations. A short time (e.g., 2 min in our setting) might prevent SPs from conducting a thorough evaluation.

8) **Adapting SPs’ training to the new stations for the assessment of communication competencies**

Participants expressed concern that the introduction of the scenarios in which SPs have to portray pre-determined verbal and non-verbal emotional hints at a pre-defined level of intensity would increase the complexity of the SP’s role-play and training. The standardization imperatives of the OSCE require SPs to provide the same information to each candidate, regardless of the quality of their communication competencies. Hence, some participants suggested allowing greater flexibility in the SPs’ role-plays. Even if the role-play and the SP-training are challenging, “*standardization can be ensured by providing SPs with examples of how to respond to “unsatisfactory, intermediate, and good” responses by students/candidates”* and train them to react differently to such behaviors.

### How can the raters’ training be improved?

9) **Adapt the raters’ training to the stations dedicated to the assessment of communication competencies**

With the introduction of OSCE stations dedicated to communication competencies, raters’ training must address all aspects related to the assessments conducted at such stations. In particular, raters should know how to use specific assessment criteria (e.g., those inspired by the Verona Coding Definitions of Emotional Sequence) and models, especially if they are not communication experts. As mentioned previously, frequent use of the same rating scale for the clinical skills component of the FLE throughout the undergraduate curriculum could also be considered as a type of training for raters. Finally, participants emphasized the importance of keeping rating scales as simple and intuitive as possible, thereby simplifying the raters’ training.

## Discussion

Our symposium identified significant elements for improvement, mainly concerning the design and development of OSCE cases and the assessment instruments.

Quality in the assessment of physician-patient communication competencies at the Swiss FLE has so far been ensured by at least three elements: the blueprint of OSCE stations providing a large sample of clinical situations (emergency, acute, chronic, and palliative) in different clinical settings (hospital or ambulatory) and disciplines, the systematic assessment of general communication competencies at each OSCE station and the monitoring of psychometric properties of the rating tool over the years [9]. This, however, should not be considered as a sufficient condition for the thorough evaluation of communication competencies.

Our suggestion to introduce concrete elements in OSCE scenarios, so as to allow candidates to be exposed to specific communication aspects (Suggestions 1 and 2) is corroborated by a recent analysis by the National Board of Medical Examiners (NBME) which reviewed all components of the United States Medical Licensing Examination (USMLE) [23]. This analysis showed that in OSCE scenarios, the biomedical content dominated over the psychosocial or emotional details. The latter, although present, often lacked sufficient detail to elicit the desired communication skills. This may partly explain why examinees focus more on data gathering than on patient-centered behaviors [23]. This supports the importance of developing OSCE scenarios linked with concrete station’s endpoint [13, 14]. For example, De Haes and colleagues developed a very interesting framework, named the *Six-function model of medical communication* [11], which provides opportunities to focus on (1) fostering the relationship, (2) gathering information, (3) information provision, (4) decision making, (5) enabling disease and treatment-related behavior, and (6) responding to emotions. For each of these functions it is possible to use validated, specific communication models, e.g. the SPIKES model for breaking bad news [27], or the Verona Coding Definitions of Emotional Sequence (VRCoDES) [25, 26]. The VRCoDES was developed to analyze emotional communication during patient encounters, and it classifies clinicians’ responses to patients’ cues and emotions into two main dimensions: *explicitness* of the response (explicitly or not explicitly referred to as the cue/concern) and *provision of space* (the response provides or reduces space for further disclosure of the cue/concern) [25, 26]. Zhou and colleagues successfully applied the VRCoDES in the context of OSCEs with good reliability [28]. To ensure validity and transparency of the assessment criteria for communication, models should: (a) be validated models, (b) be used during training, and (c) involve communication experts in case development (Suggestions 3). This prompted the idea of creating a platform to facilitate communication among the instructors of the five medical schools to exchange information and impressions about the models used in the teaching process.

Concerning the importance of ensuring authenticity in the SPs’ portrayals and the validity of the assessment, we already develop scenarios inspired by real patient narratives [27] which we enrich with psychosocial details [23]. The interesting suggestion is to enable SPs to vary their portrayals in response to whether or not the examinee adopts the expected communication attitude (Suggestion 4). Standardization of the biomedical content of cases can be maintained by providing examples of adequate and inadequate responses to emotional cues in scenarios to SPs and raters in training. Therefore, the training of SPs and raters requires attention to the balance between standardization and authenticity (Suggestions 8 and 9).

Another condition evoked to ensure validity of the assessment of communication competencies, is the selection of an appropriate rating tool (Suggestion 5 and 6). There is an ongoing debate in the literature in relation to the topic of which type of rating tool best suits the assessment of communication competencies in the OSCE setting. Recent research has suggested some advantages of using global rating scales over checklists, such as greater internal consistency [15, 29, 30] and the ability to capture multiple aspects of performance when used by experienced clinicians with adequate training [15, 31–33]. On the other hand, checklists seem to be better at capturing specific details of communication behaviors, to be less prone to rater bias, and useful to non-experts [31, 34].

Our suggestion is consistent with the finding that combining the global rating scale and checklists increases the reliability and content validity of the communication-competencies assessment [15, 22, 29, 30].

As for the appropriateness to keeping the Analytic Global OSCE Ratings [19, 20] (Suggestion 6) for the assessment of general communication competences in every station, we did not find a more suitable tool in the literature, nor did existing alternatives have advantages from a psychometric point of view [8, 16, 17]. Moreover, the alternative tools were lengthy, with 10 to 36 items [16], and hence, unsuitable in a setting where examiners complete rating scales while the OSCE encounter is in progress and the time between the two examinees is only 2 min. The Analytic Global OSCE Ratings, with its four items, seems to be the best fit for our FLE context, in terms of reliability, feasibility, and acceptance [10, 19, 35].

Our suggestions is that a well-suited evaluation tool for communication-competencies assessments in the licensing OSCE setting should be a validated scale with good psychometric properties, which is well known and accepted by raters, feasible in the exam setting, with dimensions that are consistent with instruction, and items that are able to capture the communication goals of the station.

### Limitations

The symposium was not aimed nor empowered to produce consensus statements. Indeed, agreement about the results of the thematic analysis was obtained only from the authors of this article and not from the other participants. Further, the results have not been additionally reviewed by external experts. That is why we thought it appropriate to talk in terms of suggestions and not recommendations. However, we believe that our conclusions are credible for the following reasons. Firstly, all the authors actively participated in the symposium and were therefore able to capture the essence of the discussions. Secondly, the material was analyzed separately by different authors and comes from original and verifiable sources such as video recordings of the plenary discussions. Thirdly, in the analysis we also took into account the individual “take-home massages” of each participant. Such a symposium can provide major directions; however, detailed concepts have to be developed based on these proposals. Nevertheless, to our knowledge, this is one of the few publications of this type, and the results should be useful to all professionals involved in the assessment of communication skills.

## Conclusion

This article offers nine practical suggestions, at both structural and process levels, to improve the assessment of communication competencies in high-stakes licensing OSCEs. Further research is needed to test to what extent the implementation of all these suggestions will effectively contribute to improvements in the quality of communication-competencies assessments.

## Data Availability

The datasets analyzed during the current study are available from the corresponding author on reasonable request.

## Abbreviations

OSCE(s): Objective Structured Clinical Examination(s)
FLE: Federal Licensing Exam
SP(s): Standardized patient(s)
VRCoDES: Verona Coding Definitions of Emotional Sequence
